# Interface Pressures Derived from a Calibrated Bandage Applied for Compression Therapy

**DOI:** 10.3400/avd.oa.24-00103

**Published:** 2025-01-01

**Authors:** Kotaro Suehiro, Hitoshi Sakuda, Takasuke Harada, Yuriko Takeuchi, Takahiro Mizoguchi, Ryunosuke Sakamoto, Hiroshi Kurazumi, Ryo Suzuki, Kimikazu Hamano

**Affiliations:** 1 Department of Surgery and Clinical Science, Yamaguchi University Graduate School of Medicine, Ube, Yamaguchi, Japan; 2 The Committee of Elastic Stocking and Compression Therapy Conductor of the Japanese Society of Phlebology, Tokyo, Japan

**Keywords:** compression therapy, calibrated bandage, interface pressure

## Abstract

**Objectives:** We sought to clarify the interface pressure (IP) and its variation by applying Biflex16, a calibrated bandage, to the lower leg.

**Methods:** In Study I, 50 participants applied a bandage to the lower leg of a single subject in two ways: first, with 50% overlap, while the calibration rectangle became a square (Application 1), and then with 50% overlap without intentional stretch (Application 2) which served as a control. In Study II, another 51 participants applied the bandage to their lower leg via Application 1. The IP was measured at the level of the transposition of the medial gastrocnemius muscle into the Achilles tendon (B1).

**Results:** In Study I, the median IP (37 mmHg) and interquartile range (IQR; 9 mmHg) in the standing position were the same for Applications 1 and 2. In Study II, the obtained IP and IQR values were 38 and 12 mmHg, respectively, in the sitting position. This IP was similar to that obtained in Study I, and no correlation was found between IP and leg circumference.

**Conclusions:** The variation in the IP obtained by the calibrated bandage was reasonably small when applied via Application 1. The obtained IPs did not correlate with the leg circumference.

## Introduction

Compression therapy is an indispensable treatment for chronic venous insufficiency and lymphedema. To manage various symptoms, the recommended interface pressure (IP) is indicated for each specific symptom.^1^^,^^2)^ In other words, reasonable IP control is crucial to manage such symptoms. However, obtaining a stable IP when bandages are used is technically challenging. This is mainly because the degree of stretching varies widely depending on the skill of the bandagers. To stabilize IP using bandages, the use of a calibrated bandage is an option. The target IP can be obtained by arranging the shape of the marks printed on the bandage and the width of the overlap. However, the skill of each bandager still affects the IPs, even when using a calibrated bandage. Moreover, the IP should vary according to the circumference of the limb based on the modified Laplace’s law: IP (mmHg) = tension (kgf) × number of layers × 4620/circumference (cm) × bandage width (cm).^3)^ In this study, we investigated the range of IP variation obtained using a calibrated bandage and the factors affecting this variation.

## Materials and Methods

This prospective, two-phase study was approved by the Institutional Review Board of Yamaguchi University Hospital (Center for Clinical Research, Ube, Yamaguchi, Japan, 2023-150 for Study I and 2023-216 for Study II). The participants provided written informed consent prior to enrollment. In both studies, the same calibrated bandage (Biflex16, Thuasne SAS, Levallois-Perret, France; width, 8 cm; length, 4 m; and elasticity, 125% at 10 N/cm width) was employed.

### Study I

**Figure 1:**
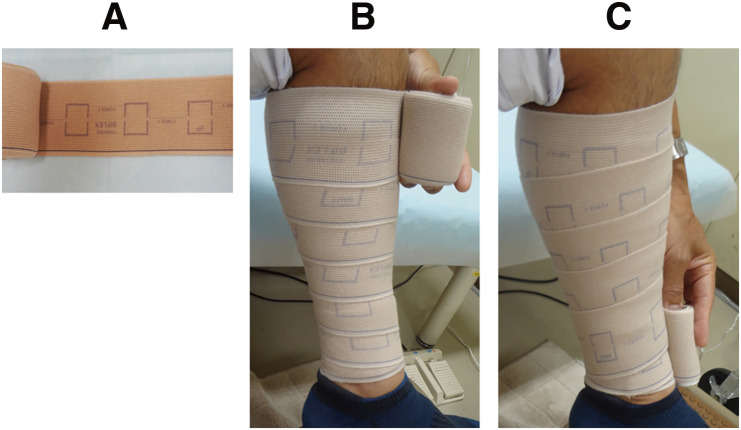
Fig. 1 Application techniques of a calibrated bandage. (**A**) Calibrated bandage (Biflex16). (**B**) Application 1. A calibrated bandage was applied from the ankle to the popliteal fossa in a spiral manner with 50% overlap, while the calibration rectangle became a square. (**C**) Application 2. The same bandage was applied from the ankle to the popliteal fossa in a spiral manner with 50% overlap, without intentional stretching in the two layers. Note that the shape of the calibration rectangle remained almost unchanged in Application 2.

In Study I, we evaluated the variation in IP caused by the bandager’s skill. The participants who applied bandages were 50 volunteers who were not familiar with bandaging (14 physicians, 15 nurses, and 18 other staff members, including clerks in our hospital) and three lymphedema therapists who were not experienced in handling the currently employed bandage. A male volunteer whose leg was wrapped with bandages was also enrolled. His leg circumference was 40.5 cm at maximum calf level, 29.1 cm at B1 level (the level of transposition of the medial gastrocnemius muscle into the Achilles tendon), and 22.8 cm at the ankle level. Following a 2-min guidance, the participants applied bandages to the lower leg of a single subject, that is, a male volunteer, using the following two methods (**Fig. 1**):

Application 1: The bandage was applied spirally from the ankle to the popliteal fossa with a 50% overlap, while the calibration rectangle became a square.

Application 2: The bandage was applied in a spiral manner to the same area with a 50% overlap in two layers, namely, going back and forth, for comparison. In this case, the bandage was applied only to fit the leg shape without intentional stretching, as previously reported.^4)^

In a preliminary study, we confirmed that the median IP obtained by Application 1 was approximately 35 mmHg in the standing position and that Application 2 would provide a similar IP.

The IP was measured immediately after the application of the bandage using an air pack-type analyzer (Model AMI-3037-SB; AMI Techno Co., Ltd., Tokyo, Japan). The sensor was attached to the medial aspect of the lower leg at the B1 level.^5)^ Following each bandage application, the IP was measured while the subject was in the supine position and then in the standing position. The stiffness of the applied bandage was determined using the static stiffness index (SSI), which is defined as the difference between the IP in the supine and standing positions (mmHg).^6)^

### Study II

Study II evaluated the impact of leg size/shape and bandagers’ skill on the variation in IPs. The participants included 51 individuals who attended a standardized education and training course for compression therapy providers held by the Committee of Elastic Stocking and Compression Therapy Conductors of the Japanese Society of Phlebology. Among these, 45 participants were not familiar with bandaging (3 physicians, 40 nurses, and two physiotherapists), and six nurses were trained in bandaging. The leg sizes of the participants are summarized in **Table 1**. The participants learned how to use the calibrated bandage using a 2-min video and then applied the bandage to their own lower leg using Application 1. For convenience, the IP was measured only in the sitting position at the B1 level.

**Table:** Table 1 The leg sizes of the participants in Study II

Level	Circumferences (cm)
Calf	34 (30–41)
B1	24 (21–32)
Ankle	20 (18–23)

Calf: the level of the calf of its maximum diameter; B1: the level of transposition of the medial gastrocnemius muscle into the Achilles tendon; ankle: the level of the upper edge of the malleolus

### Statistical analysis

Unless otherwise indicated, the results are expressed as median (range) or count. The Wilcoxon signed-rank sum test was used to test the changes in IPs within the same participants. The Mann–Whitney *U*-test was used to test the measurement differences among participants. Correlations between the IPs and leg circumference were tested using linear regression analysis. Statistical analyses were performed using JMP 11.0 (SAS Institute, Cary, NC, USA). Statistical significance was set at *p* <0.05.

## Results

### Study I

**Figure 2:**
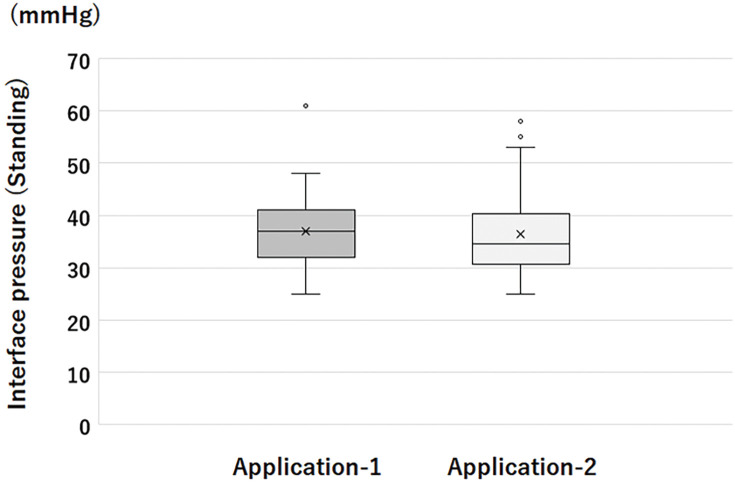
Fig. 2 The interface pressures obtained by Applications 1 and 2.

The IPs obtained using Applications 1 and 2 are shown in **Fig. 2**. When the bandage was applied using Application 1, the IP obtained was 26 mmHg (17–47 mmHg) in the supine position and 37 mmHg (25–61 mmHg) in the standing position. The SSI was 11 mmHg (6–18 mmHg). The manufacturer advocates that an IP of 21–25 mmHg can be obtained by Biflex16 in the supine position when applied using Application 1. However, the obtained IPs were higher than those of 25 participants (50%) in the supine position. When the bandage was applied using Application 2, the IP obtained was 21 mmHg (12–49 mmHg, *p* <0.001 vs. Application 1) in the supine position and 37 mmHg (25–58 mmHg, *p* = 0.39 vs. Application 1) in the standing position. The SSI was 16 mmHg (8–22 mmHg, *p* <0.0001 vs. Application 1). The IPs’ interquartile ranges (IQR) in the standing position were the same (9 mmHg) in Applications 1 and 2.

### Study II

**Figure 3:**
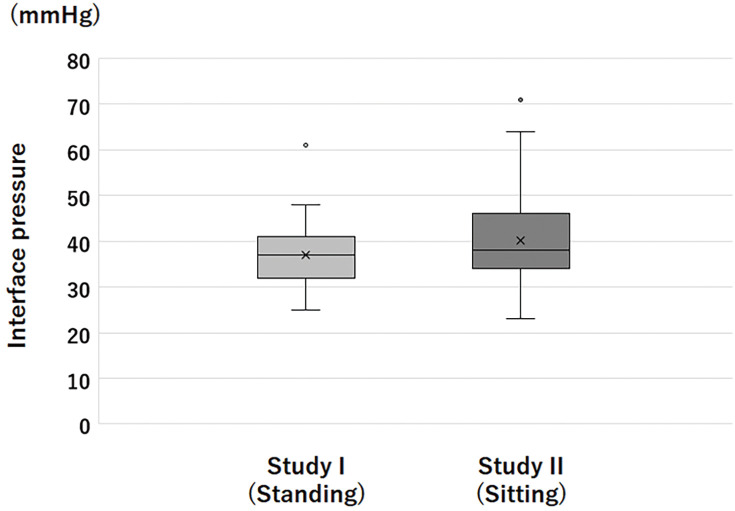
Fig. 3 The interface pressures obtained by Application 1 in Study I and Study II. Note that the interface pressures were measured in a standing position in Study I, while that was measured in a sitting position in Study II.

**Figure 4:**
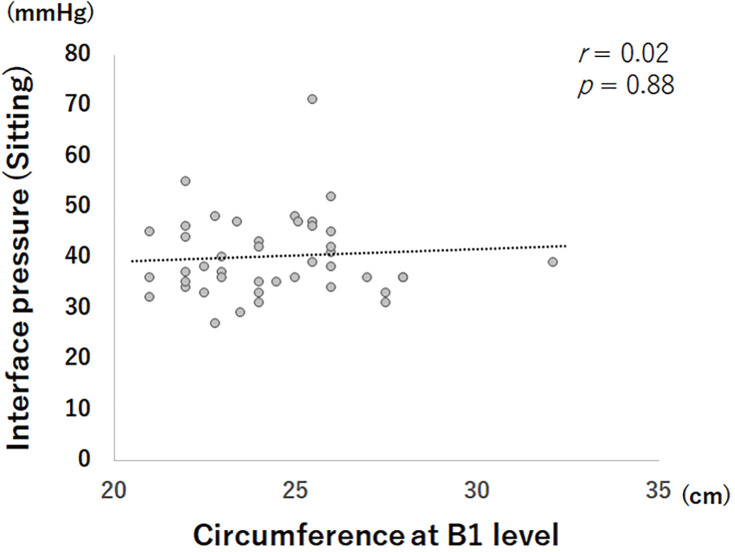
Fig. 4 The correlation between the interface pressures and leg circumferences measured at the level of B1.

The IPs obtained using Application 1 in Study I and II are shown in **Fig. 3**. In Study II, the obtained IP was 38 mmHg (23–71 mmHg) in the sitting position, and the IQR was 12 mmHg. This IP was similar to that obtained in Study I despite being measured in the sitting position. This IQR was slightly higher than that observed in Study I. The IPs obtained in Study II did not show a significant correlation with circumference at the B1 level (**Fig. 4**). Outliers, defined as <20 mmHg or >60 mmHg in this study, were found in 4 participants (8%). All participants had an IP >60 mmHg, and all of the causes were overextension of the bandage. An IP <20 mmHg was not found in this series.

## Discussion

### IP and its variation derived from a calibrated bandage

Benigni et al.^7)^ previously reported an IP obtained using Biflex16. When the bandage was applied with a 30% stretch (calibration to square) and 50% overlap, which was the same manner as Application 1 in this study, the obtained IP was 26 mmHg (23–29 mmHg) in a supine position. They also applied the same bandage in the spica, in turns of the 8-technique, which essentially doubled the number of layers, without intentional stretching. This provided an IP of 23 mmHg (16–30 mmHg) in the supine position. These IPs were similar to those obtained in Study I. Therefore, excellent reproducibility of achieving the target IP using Biflex16 was confirmed.

In Study I, various bandagers applied the bandage to the same leg. Therefore, the variation in IPs was considered to be solely due to the bandager’s skill. The variation in IP in Application 1, as assessed using IQR, was 9 mmHg, which suggested that variation in bandage skills would still cause variation in IP in this range. We previously reported that the technique used in Application 2 caused a small variation in the IPs.^4)^ In Study I, the IQRs were the same for Applications 1 and 2. Accordingly, the variation in the IP obtained using Application 1 was considered reasonably small. It can also be concluded that the application of Biflex16 by Application 1 could provide the intended IP stably, even when handled by inexperienced bandages.

In Study II, in which the bandagers applied the bandage to their legs, the IQR was 12 mmHg, which was slightly higher than that observed in Study I. This suggests that the variation in leg size and/or shape further affects the variation in IP. Outliers (<20 mmHg or >60 mmHg) were found in 8% of the participants in Study II. Considering that all of the causes of the outliers were overextension of the bandage and no cases of <20 mmHg were found, the bandagers tended to stretch the bandage too much when Biflex16 was used.

### Correlation between IPs and leg circumferences

Sub-bandage pressure is generally believed to follow modified Laplace’s law.^3)^ Namely, the IP is assumed to be lower when the bandage is applied to a larger leg. However, no such correlation between the IPs and circumferences measured at the B1 level was confirmed in the current study. Similar results have been previously reported by Garrigues-Ramón et al.^8)^ They explained that this was largely caused by anatomical components such as musculoskeletal and/or soft tissue variables, which act as compression-damping forces. Their report also stated that the obtained IP values still showed the expected results according to the manufacturer. By contrast, in this study, the IP obtained using Application 1, that is, 26 mmHg (17–47 mmHg) in the supine position, was higher than that advocated by the manufacturer (21–25 mmHg in the supine position). This may be because the participants tended to stretch the bandage too much, as described above.

Using Application 1, the median IP in the standing position was 37 mmHg in Study I, whereas that in the sitting position was similar (38 mmHg) in Study II. However, the IP in the sitting position should be lower than that in the standing position. This result could be explained by the fact that the leg circumference of a single volunteer in Study I was larger than the median leg circumference of the participants in Study II if the modified Laplace’s law was applied. However, this result may also be due to the effects of the anatomical components of the participants, as described above. These results should be further investigated in future studies.

### Limitations

First, measuring all IPs in the same setting regarding the participants and body positions was ideal. However, owing to the difficulty in gathering volunteers, we had to collect data from two separate studies, which hindered us from obtaining definite conclusions. Second, most participants in both Studies I and II were beginners and were not familiar with bandaging techniques, which could have affected the variation in IPs. However, whether such variations could be minimized if the participants were trained is uncertain and should be clarified in future studies. Third, the level of B1 is not a specific line but a zone. Therefore, the circumferences in the upper and lower areas of the B1 level differ by a few centimeters. Because many participants in this study were not familiar with circumference measurement, the level of circumference might have differed slightly, which could have affected the results. Fourth, the IPs were measured using five air-pack-type analyzers of the same model. However, measurement errors among these analyzers were not verified, which might have affected the results. Fifth, the IPs were evaluated only in the static state immediately after the application of the bandage in this study. However, IPs in the dynamic state as well as the changes in the IPs over time should be elucidated in future studies to reveal the characteristics of the bandage further.

## Conclusion

The calibrated bandage employed in the current study showed excellent reproducibility in providing the target IP with a reasonably small variation, even when used by beginners. The obtained IPs tended to be higher than those advocated by the manufacturer and showed no correlation with the circumference.

## Declarations

### Declaration of conflicting interests

The authors declare that there is no conflict of interest.

### Acknowledgments

None.

### Funding

This research did not receive specific grants from any funding agency in the public, commercial, or not-for-profit sectors.

### Author contributions

Study conception: KS

Data collection: KS, HS, TH, YT, TM, RSa, HK, and RSu.

Analysis: KS

Investigation: KS and HS

Writing: KS and HS

Funding acquisition: None.

Critical review and revision: all authors

Final approval of the article: all authors

Accountability for all aspects of the work: all authors.
